# Altered Mental Status and a Not-So-Benign Rash

**DOI:** 10.1155/2011/684572

**Published:** 2011-10-19

**Authors:** Aakash N. Bodhit, Latha Ganti Stead

**Affiliations:** ^1^Department of Emergency Medicine, University of Florida, Gainesville, FL 32610, USA; ^2^Division of Emergency Medicine Research, Department of Emergency Medicine, University of Florida, Gainesville, FL 32610, USA

## Abstract

*Introduction*. The authors are presenting a case of Thrombotic Thrombocytopenic Purpura (TTP) that presented with complaints of altered mental status and found to have petechiae. *Case Presentation*. An 81-year-old female patient presented to the Emergency Department (ED) of a tertiary care hospital with chief complains of dizziness, slurred speech, and weakness. She was found to have lower extremity petechiae on physical examination. On blood exam, she had thrombocytopenia, and her peripheral blood smear showed schistocytes. Her renal function was also impaired. The CT scan of head was without any abnormality. She was finally diagnosed as having TTP and transferred to ICU but ultimately passed away. *Conclusion*. TTP is a rare syndrome with preventable mortality if diagnosed early and managed appropriately with plasmapheresis. The Emergency Department physicians should be aware of the presenting symptoms and signs of TTP.

## 1. Introduction

Altered mental status (AMS) can be caused by a myriad of conditions. Patients with AMS are common in the Emergency Department, and a significant number of them require timely intervention as many of the underlying conditions are treatable [1]. When a patient presents to the emergency department with neurological symptoms such as altered mental status, seizures, hemiplegia, paresthesias, visual disturbance, or aphasia along with a petechial rash, the patient may be suffering from an uncommon syndrome called Thrombotic Thrombocytopenic Purpura (TTP). It was the cause of clinical presentation in this case. It is a rare condition with incidence of 4 to 11 cases per million annually [2]. Though it is associated with some conditions, the exact etiology is unknown. It is important to take TTP in consideration in differential diagnosis, because if diagnosed early and managed properly, the mortality is reduced to 10–20% compared to nearly 90% mortality when not diagnosed earlier and managed properly. The sequelae are not common in patients who survive.

## 2. Case Presentation

**Figure 1:**
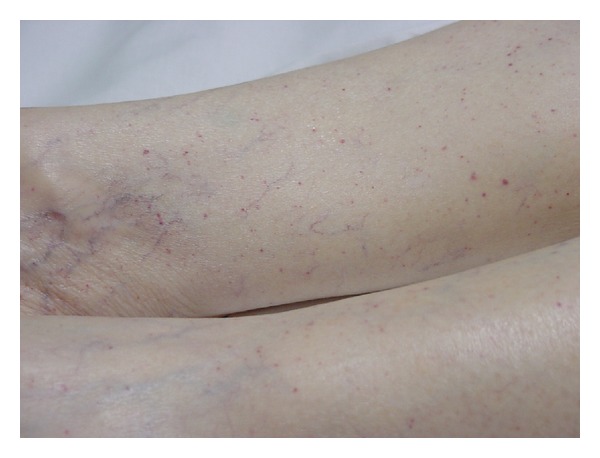
Typical petechial rash on patient's lower extremities.

An 81-year-old female presented to the Emergency Department of a tertiary care hospital at around 03:15 p.m. one day. She was in her normal state of health until 02:15 p.m. when she developed dizziness, weakness, and slurring of speech at her nursing home. She had a history of dementia but reportedly was able to manage her activities of daily living. On the morning of that day, she needed assistance with getting to the bathroom. The patient also had complained of weakness on that day, and the nursing home doctor had held her beta-blocker medication after that complaint. In the emergency room, she was found to have a nonfocal neurological examination. The vitals were normal except for increased pulse rate. Remainder of the physical exam was remarkable for lower extremity petechial rash (Figure 1).

**Figure 2:**
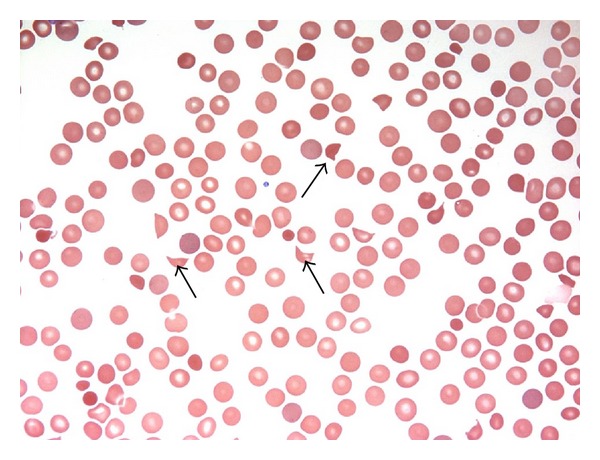
Peripheral smear of the patient demonstrating schistocytes (arrows).

The Emergency Department evaluation revealed a head CT without any acute pathology. The EKG showed sinus tachycardia with inverted T-waves in the lateral leads. Study of cardiac enzymes showed elevated Troponin T level (0.6 ng/mL (normal: <0.03 ng/mL)) and elevated CKMB level (54.9 ng/mL (normal: <6.2 ng/mL). The platelet count was extremely low (6 × 10^9^ (normal: 150–450 × 10^9^)). The RBC count and WBC count were within normal limits. The peripheral blood smear showed schistocytes (Figure 2). The patient had prolonged bleeding time. Results of basic metabolic profile were unremarkable. Urine dipstick measurement showed presence of protein (3+) and blood (3+).

## 3. Diagnosis

After a consultation with the Neurology Department in the ED, working diagnosis of Thrombotic Thrombocytopenic Purpura (TTP) was made after ruling out more common causes of altered mental status such as infectious meningitis, CVA, and metabolic or electrolyte abnormalities. The patient was transferred to the Medical Intensive Care Unit, where she died 9 hours later.

## 4. Discussion

Altered mental status is a common presenting symptom in the Emergency Department population, and the differential diagnosis is very broad. The common causes should be considered first depending upon the patient characteristics. If those have been ruled out, further management should be focused on finding out one of the not-so-common culprits.

The patient described in this case was elderly demented patient. In such patients, it is necessary to take into consideration the baseline mental status when evaluating for altered mental status. Common causes for altered mental status in such patients are CVA, infectious meningitis, renal failure, and metabolic abnormalities. The patient in question did not have fever, had normal basic metabolic profile and CT scan and normal differential count, but had thrombocytopenia along with abnormal renal function. The presence of schistocytes on peripheral blood smear was responsible for confirmation of diagnosis of TTP.

This case illustrates the following main teaching points about TTP.

- The classic pentad of TTP consists of (1) fever, (2) anemia, (3) thrombocytopenia, (4) renal insufficiency, and (5) neurologic dysfunction. An easy mnemonic for this is “FAT RN [3].” This classic pentad is present in less than 20% of patients. Patients most commonly have 3 of the 5 traits, as did our patient (thrombocytopenia, renal insufficiency, and neurologic dysfunction).
- Administration of platelets in an effort to correct the thrombocytopenia, while seemingly intuitive, can be fatal. The mainstay of treatment is plasmapheresis, and *not *replacing platelets.
- The reason it is important to consider TTP in any patient presenting to the ED with an acute change in mental status is because, when diagnosed promptly, 80% of patients survive. When the diagnosis is missed, 80% of patients die because of it [4].

After the introduction of plasmapheresis as the effective treatment, the diagnosis of TTP is done mostly on the basis of microangiopathic hemolytic anemia and thrombocytopenia, as it allows for easier diagnosis and faster treatment. Additionally, all the traits of classic pentad are not present always.
